# Assessment of Perceived Knowledge and Beliefs Toward Cancer Immunotherapy Among Regional Healthcare Providers

**DOI:** 10.7759/cureus.106093

**Published:** 2026-03-29

**Authors:** Amal Ismail, Alaa A Alnasr, Bushra S Alamer, Ghada Albahli, Raniya H Alharbi, Sara Alsawyan, Eiman S Ibrahim

**Affiliations:** 1 Pharmacy Practice, Unaizah College of Pharmacy, Qassim University, Unaizah, SAU; 2 Pharmacy Practice, Unaizah College of Pharmacy, Qassim University, Uniazah, SAU; 3 Pharmacy Practice, Unaizah College of Pharmacy, Qassim University, Buriadah, SAU

**Keywords:** awareness, cancer immunotherapy, cytokines, healthcare providers, immune checkpoint inhibitors, qassim, saudi arabia

## Abstract

Background

Cancer immunotherapy represents a rapidly advancing frontier in oncology. Its increasing integration with established treatment modalities underscores the necessity for healthcare providers (HCPs) to be well-informed. Awareness among HCPs is critical for effective multidisciplinary collaboration and optimal patient care. This study aimed to assess HCPs' awareness and perceptions of cancer immunotherapy.

Methods

A cross-sectional study was conducted using a self-administered questionnaire among HCPs in the Qassim region of Saudi Arabia between November 2022 and March 2023. The survey evaluated perceptions and self-reported knowledge regarding cancer immunotherapy and its associated side effects.

Results

Among 374 participants (mean age: 41 years; 62% male, 38% female), 35% had ≥10 years of professional experience. Overall knowledge was low, with 258 participants (69%) scoring poorly. A majority (201, 53.7%) considered chemotherapy the best treatment option, while 159 (42.5%) perceived immunotherapies as the most expensive modality. A significant knowledge gap was evident, with 269 HCPs (71.9%) reporting a lack of familiarity with immunotherapy and 163 (43.6%) expressing uncertainty about its effectiveness compared to other therapies. The need to address this knowledge deficit was affirmed by 304 respondents (81.3%).

Conclusion

This study reveals poor perceived knowledge of cancer immunotherapy among HCPs in the region, a prevalent preference for chemotherapy as the optimal treatment, and a common perception of immunotherapy as costly. To bridge this gap, we recommend promoting HCP engagement in specialized field workshops and updating educational curricula, as better knowledge will increase appropriate use and improve outcomes, which strengthens policy support and public trust.

## Introduction

Over recent decades, extensive research in oncology and pathology has significantly improved our understanding of the molecular pathways of tumour cell proliferation, therapeutic resistance, metastatic dissemination, and immunological evasion. This resulted in the development of drugs that can selectively target tumour cells. Currently, immunotherapy represents a highly promising treatment option for systemic cancer [1].

Immunotherapies are divided into passive immunotherapy, which includes anti-cancer vaccines and adoptive T-cell transfer (CAR T-cells), and active immunotherapeutic options like monoclonal antibodies, immune checkpoint inhibitors (ICIs), and cytokines (interferons and interleukin-2). Immune checkpoint inhibitors aim to restore and enhance cytotoxic T-cell anti-tumour function. They do this by interfering with inhibitory signals that make T-cells less active, either by inhibiting immune cell receptors on T-cells or by binding to ligands on antigen-presenting or tumor cells [2].

Several factors can influence the effect of tumour immunotherapy. First, it is related to human immunity, which is closely linked to genetics and internal microbiota. The number and type of gut flora influence not only cancer incidence but also chemo- and immunotherapy sensitivity. These findings are expected to be used in clinical practice to improve immunotherapy efficacy by changing patients' lifestyles or supplementing gut flora. Furthermore, consuming antibiotics has a negative impact on the balance of intestinal flora, resulting in lower immunotherapy efficacy; as a result, antibiotics should be avoided during immunotherapy.

Second, patients with low intra-tumour heterogeneity of tumor neoantigens and a large number of clonal neoantigen sources have more therapeutic benefits. Third, it is linked to things like daily routine, food, bacterial illness, and drug use [3]. Patients' responses to immunotherapy can be assessed by detecting certain biomarkers, such as tumor mutation burden (TMB), gene expression profiles, and DNA methylation profiles. Understanding tumor biology and mutations is crucial for determining the immune response and developing new biomarkers. This knowledge can improve the personalization of treatment strategies for patients, leading to better outcomes [4]. Clinical predictive models based on biomarkers will lead to effective clinical plans. In patients with non-small cell carcinoma, the selection of immune checkpoint inhibitors depends on the expression of programmed death (PD)-L1 and the high TMB, while in patients with colon cancer, combining TMB with microsatellite instability (MSI) and deficient mismatch repair (dMMR) improves the response prediction [5-8]. Homologous recombination deficiency (HRD) responds to therapies like poly ADP-ribose polymerase inhibitors (PARP inhibitors) because these tumors accumulate more genetic instability, creating a higher mutational burden that results in the generation of neoantigens. Studies indicate that tumors with a high mutational load respond more effectively when combined with ICIs, as this combination enhances the immune system’s ability to detect and destroy cancer cells [9].

Although cancer immunotherapy is perceived as a considerable enhancement to the current treatment options for its safety profile, side effects of monoclonal antibodies can include hypotension, development of cardiomyopathy, congestive heart failure, and life-threatening arrhythmias, while ICIs primarily induce autoimmune symptoms like myocarditis and pericarditis. Treatment with Blinatumomab can lead to cytokine release syndrome and neurotoxicity, which can occur with or without cytokine release syndrome (CRS) due to the mechanism by which blinatumomab induces T-cell adherence to endothelial cells [10,11]. As the field of cancer immunotherapy is complex and rapidly evolving, healthcare professionals (HCPs) involved in the care of patients with cancer need to understand immunotherapy so that they can interpret the growing body of clinical data and make optimal use of immunotherapies alongside the established treatment modalities [12].

The European HCPs involved in cancer care answered a survey assessing their knowledge and experience regarding this novel therapeutic option, and the participants showed that they had a high level of awareness but minimal direct experience. The study concluded that more educational initiatives are needed, with a focus on the sharing and interpretation of long-term clinical data, as well as a better understanding of the mechanisms of action of various cancer immunotherapies It was shown that the majority (60%) of all respondents indicated they had an overall positive attitude toward cancer immunotherapeutics/ therapeutic cancer vaccines (TCVs), although there was considerable variation across the HCP specialities. A negative attitude was reported by only 3% of the HCPs [13].

In Saudi Arabia, multidisciplinary HCPs in eastern Saudi Arabia had a low level of awareness and grasp of the principles of cancer immunotherapy. They concluded that, in addition to developing new cancer immunotherapy treatments, HCPs must be educated in the basic science of immunotherapy. A follow-up survey of HCPs working in cancer care is required [14,15].

Health providers are working as a team, playing different roles in treatment plan selection, including initial assessment, pre-treatment evaluation, drug selection, safe administration, monitoring of side effects, and patient education. A strong scientific foundation makes it easier for HCPs to stay current and adapt to emerging evidence. This study aimed to assess the perceived knowledge and beliefs of healthcare workers regarding cancer immunotherapy.

## Materials and methods

A quantitative cross-sectional study was conducted at four general hospitals in the Qassim region of Saudi Arabia among healthcare professionals, including physicians, pharmacists, nurses, and laboratory technicians, across various departments of the selected hospitals, with data collected over a three-month period. The participants in this study were licensed healthcare professionals from Saudi Arabia and abroad who were present at the hospital during the data collection period. They had been affiliated with the institution for a minimum of one year and had consented to engage in the self-administered questionnaire that was distributed. 

Questions were adopted from previous literature [12,14] and were further validated by experts' revisions after the application of a pilot study, the outcomes of which were excluded from the final analysis. Cronbach's alpha test for internal consistency was applied and showed good internal consistency of the questionnaire questions. The questionnaire was composed of three sections for the determination of participants' general characteristics, followed by running a knowledge test. Knowledge was assessed by using a self-perceived knowledge scale, where participants rated their understanding of cancer immunotherapy concepts as low, moderate, or high. The self‑knowledge assessment evaluated participants’ perceived understanding of cancer immunotherapy across several core domains. Items asked healthcare providers to rate their confidence in their general understanding of immunotherapy, its mechanism of action, and the clinical indications for which it is used. Additional items assessed perceived ability to select appropriate patients, recognise and manage immune‑related adverse events, and differentiate immunotherapy from conventional chemotherapy in terms of effectiveness and clinical role. Participants also rated their familiarity with relevant clinical guidelines and their awareness of cost and access considerations. Together, these items provided an overview of respondents’ self‑perceived readiness to discuss and utilise immunotherapy in clinical practice. The full questionnaire can be viewed in the Appendix 1. 

The sample size was calculated using the electronic Raosoft software calculator version 2.3 (Raosoft Inc., Seattle, USA). At a 5% margin of error and a 95% confidence interval, the representative sample size was 374 or more. Stratified random sampling was from randomly selected hospitals, and the health care providers were divided into strata (doctors, nurses, pharmacists, laboratories, and others). Data collection was conducted during working hours throughout different daily shifts. Sampling was conducted after ethical approval was obtained from the local research ethics committee (QREC) under the number (1443930640).

A written informed consent was signed by healthcare workers upon understanding the purpose of the research and agreeing to participate. Data were entered into an Excel sheet (Microsoft Corporation, Redmond, USA) coded, and exported to SPSS version 25 (IBM Corp., Armonk, USA) for further processing and statistical testing. 

## Results

The study surveyed a total of 374 health professionals from the 460 invited healthcare workers, with a response rate of 81.3%, from different age groups, ranging from 25 to 55 years old, with a mean of 41 and a standard deviation of 12. More than half of the participants were male, at 62% (232). The survey was conducted at various departments: Medicine, 21.7% (81); Surgery, 7.2% (27); Paediatrics, 4.8% (18); Obstetrics and Gynaecology, 1.9% (7); and a substantial 60.7% (277) drawn from a multitude of other departments, including emergency, cardiology, radiology, intensive care, and pathology, which showed big differences in observed vs expected frequencies. 

In terms of the participants' experience, a significant proportion of participants, constituting 35%, possessed over a decade of professional experience (Table 1).

**Table 1:** General characteristics of participants (N=374). *Includes psychiatrists, dietitians, and physical therapists; **Includes: Emergency, cardiology, radiology, intensive care, pathology.

Variant	Frequency (N=374)	Percent (%)
Gender		
Male	232	62
Female	142	38
Profession		
Physician	75	20.1
Nurse	103	27.5
Pharmacist	64	17.1
Labratorist	55	14.7
*others	77	20.6
Medicine	81	21.7
Surgery	27	7.2
Pediatrics	18	4.8
Obstetrics and Gynecology	7	1.9
Pharmacy	14	3.7
**others	277	60.7
Years of Experience	
1-3	93	24.9
3-5	46	12.3
5-7	48	12.8
7-10	56	15
More than 10 years	131	35

In terms of perceived knowledge assessment, general knowledge about cancer immunotherapy was low (49.2%), and only 45.7% had acknowledged immunotherapy as an adjuvant therapy. Our survey asked several questions about different types of cancer immunotherapy. Approximately half of the participants reported knowledge about monoclonal antibodies (52.7%), but didn’t recognise the T-cell-related immunotherapy methods (52.7%).

Participants reported poor knowledge about the role of vaccination in the treatment of cancer (54.3%) and its significance in preventing infections caused by carcinogenic viruses (54.3%).

Healthcare providers involved couldn’t recognise the roles of cytokine, cytotoxic T-lymphocyte antigen (CTLA)-4 & PD-1 in cancer immunotherapy, at 54.3% and 57.8%, respectively. The significance of immuno-biomarkers as predictors of response was acknowledged by 52.1%.

**Figure 1:**
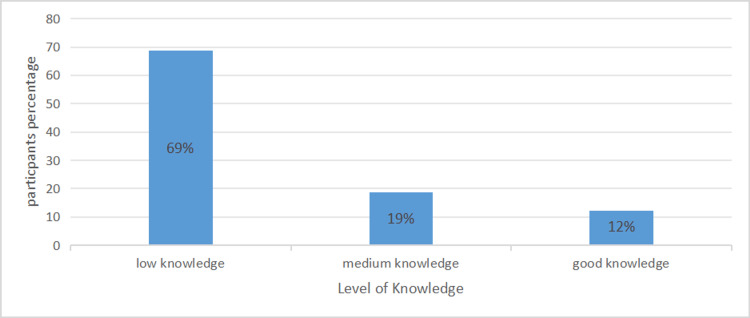
Perceived knowledge level assessment of participants (N=374).

Overall, after scoring the results of knowledge assessment questions, the results showed that 69% (258) of participants had low perceived knowledge (Figure 1).

After we applied for the normality test, non-parametric statistics (Spearman's rho) were selected for correlation. The correlation of knowledge level with profession (p=0.074) and years of experience (p=0.152) showed insignificant results (p-value <0.05). revealing no correlation with professions, regardless of the participants' number of years of experience. 

A binary logistic regression model was used to examine the effect of profession and years of experience on the likelihood of showing good knowledge on the knowledge assessment. The model explains about 25% of the variation (moderate). Neither profession nor years of experience significantly predicts the outcome.

**Figure 2:**
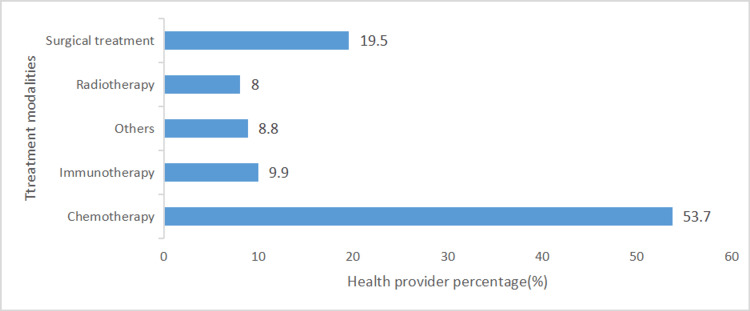
The best promising cancer therapy modality selection based on participants' perspectives (N=374).

Out of the total (374) healthcare providers, 53.7% (201) believe that chemotherapy is the most promising modality of therapy for most types of cancer, while 9.9% select immunotherapy (Figure 2).

About 42.5% (159) are convinced that cancer immunotherapy is more expensive than other cancer therapies. Many participants, 71.9% (269), agreed on the lack of information about cancer immunotherapy and approved the need for staff education about immunotherapy, 81.3% (304).

Around 34.8% (130) of participants agreed that good marketing can improve the prescription rates, and 80.2% (300) think that financial support programmes are necessary to improve access to modern immunotherapy (Table 2).

**Table 2:** Participants' perceptions regarding immunotherapy (N=374).

Participants' Perceptions N=374	yes	No	Not sure
Immunotherapy is more expensive than other cancer therapies.	42.5% (159)	14.7% (55)	42.8% (160)
There is a lack of information about cancer therapy.	71.9% (269)	8% (30)	20.1% (75)
Marketing is needed to improve the use of cancer immunotherapy compared to other treatments.	34.8% (130)	21.7% (81)	43.6% (163)
Staff education about immunotherapy is necessary.	81.3% (304)	4% (15)	14.7% (55)

## Discussion

Immunotherapy has now been clinically approved as an effective management strategy for numerous cancers. There are incredible potential synergistic combinations of drugs, and the co-administration of immunotherapy agents along with conventional cancer therapy can achieve the best treatment results [15]. 

The study aimed to assess the awareness of health professionals about the new modality of cancer therapy, as they are considered important influences consulted by patients when referred by specialists for immunotherapy as an effective treatment option. After searching the literature, we found that no similar previous study was conducted in the region, and only a few studies concerning immunotherapy have been published. Nurses were the most frequent respondents (27.5%); this was due to their participation in different departments like surgery, medicine, paediatrics, and even laboratory and radiology. Ten years of experience was found in more than a third of the participants, indicating a well-experienced group of respondents.

In terms of self-knowledge assessment of healthcare workers, our survey conducted several. This study assessed perceived rather than objective knowledge, as perceived knowledge reflects how confident healthcare workers feel about their understanding, an important factor influencing clinical decision-making, willingness to adopt new therapies, and patient counselling practices. Additionally, perceived knowledge measures impose less cognitive burden on participants, which is particularly relevant when surveying individuals from diverse educational backgrounds and specialities. Questions about different types of cancer immunotherapy and the participants' knowledge about cancer immunotherapy were low at 49.2%. Our respondents showed a low score (69%). As compared to a similar study conducted in eastern Saudi Arabia, 55% of the participants reported low knowledge [12].

The correlation of knowledge level with profession and years of experience showed negative results, agreeing with the findings of the Hamad study, yielding no significant effect of what the medical profession is and how experienced the participants are on their results. A different study conducted among physicians who delivered chemotherapy in the Arabian Gulf countries showed that none of the respondents reported a lack of awareness of immunotherapy or its indications, but 62.5% reported having limited experience in implementing the therapy [13,14,16]. 

The majority, 81.3% (304) of participants, believe that it is important to study the science of immunotherapy, similar to the Mellstedt study, in which a large proportion, 76%, of healthcare providers were interested in learning more about cancer immunotherapy.

In the current study, the perception of participants regarding cancer immunotherapy was negative, while in the Mellstedt study, the majority (60%) of all respondents indicated that they had an overall positive perception of cancer immunotherapeutics and therapeutic cancer vaccines, although there was considerable variation across the HCP specialities. A negative perception was reported by only 3% of the HCPs.

Out of the total participants, 42.5% reported that immunotherapy is more expensive than other cancer treatments, similar to Hamad's study, which reported by 44.4% of participants [12]. This can be considered a barrier for the administration of immunotherapeutic drugs in patients who are not under health insurance coverage. HCPs may feel conflicted; they want to offer effective treatments, but they also know the financial impact can be severe

This study assessed perceived rather than objective knowledge, as perceived knowledge reflects how confident healthcare workers feel about their understanding, an important factor influencing clinical decision-making, willingness to adopt new therapies, and patient counselling practices. Additionally, perceived knowledge measures impose less cognitive burden on participants, which is particularly relevant when surveying individuals from diverse educational backgrounds and specialities. Although this study was the first conducted in the region, and its results represent diverse cities while encompassing various types of healthcare providers, it also possesses several limitations. A significant barrier encountered by the researchers was the language, as most healthcare providers exhibited a preference for Arabic enquiries over English ones. Professional Arabic translation may improve knowledge scores in further studies. An additional challenge faced during the data collection phase was time constraints, as workers found it difficult to complete the questionnaires during their working hours. Furthermore, the reluctance to disclose inadequate knowledge constituted a notable limitation; many healthcare practitioners candidly admitted in personal conversations that their understanding of immunotherapy was limited, a sentiment that was not reflected in their survey responses. The absence of comparable studies addressing the objectives of interest posed a considerable obstacle for the researchers. Consequently, further investigations are warranted in other regions of Saudi Arabia to facilitate the generalisation of these findings and conduct in selected professions with objective knowledge assessment to allow more valid comparisons. 

## Conclusions

We concluded that although cancer therapy continues to advance rapidly, healthcare providers in the region demonstrated low perceived knowledge and generally negative perceptions of cancer immunotherapy. This limited understanding may undermine their confidence, reduce their willingness to adopt immunotherapy as a treatment option, and affect the quality of patient counselling. These findings highlight the need for broader, objective future studies across the region to better assess knowledge gaps and support the effective integration of immunotherapy into clinical practice.

## Appendices

Appendix 1

**Table 3:** Questionnaire.

1-General characteristics	
Age	__________________________
Gender	A. Male B. Female
Profession	A. Physician B. Nurse C. Medical laboratory scientist D. Others: __________________________
Department	A. Medicine B. Surgery C. Pediatrics D. Obstetrics & Gynaecology E. Pharmacy F. Others: …………__________________________
Years of experience	A. 1–3 years B. 3–5 years C. 5–7 years D. 7–10 years E. More than 10 years
2-Self-knowledge Assessment:	
Cancer immunotherapy knowledge	Low ☐ Medium ☐ High ☐
Immunotherapy as adjuvant therapy knowledge	Low ☐ Medium ☐ High ☐
Monoclonal antibodies (moAbs) knowledge	Low ☐ Medium ☐ High ☐
T-cell methods knowledge	Low ☐ Medium ☐ High ☐
Role of vaccines in cancer immunotherapy	Low ☐ Medium ☐ High ☐
Vaccines preventing carcinogenic virus infections	Low ☐ Medium ☐ High ☐
Cytokines in cancer therapy	Low ☐ Medium ☐ High ☐
CTLA4 & PD1	Low ☐ Medium ☐ High ☐
Immunobiomarkers as predictors of response	Low ☐ Medium ☐ High ☐
Side effects of cancer immunotherapy	Low ☐ Medium ☐ High ☐
perceptions – In your point of view:	
Best promising cancer therapy	A. Chemotherapy B. Surgical treatment C. Immunotherapy D. Radiotherapy E. Others: __________________________
Immunotherapy is more expensive	Agree ☐ Disagree ☐ Not sure ☐
Lack of information about cancer immunotherapy	Agree ☐ Disagree ☐ Not sure ☐
Good marketing for immunotherapy	Agree ☐ Disagree ☐ Not sure ☐
It is important to study immunotherapy science	Agree ☐ Disagree ☐ Not sure ☐
Financial support increases access	Agree ☐ Disagree ☐ Not sure ☐
